# Proteomics of post mortem brains in early‐ and late‐onset Alzheimer's disease: Unraveling differential Aβ effects and potential AD biomarkers

**DOI:** 10.1002/alz.71662

**Published:** 2026-07-12

**Authors:** Evangelia Thanou, Andrea Ganz, Débora Pita‐Illobre, Frank Koopmans, Jeroen J. M. Hoozemans, Annemieke Rozemuller, Henne Holstege, August B. Smit, Ka Wan Li

**Affiliations:** ^1^ Department of Molecular and Cellular Neurobiology, Center for Neurogenomics & Cognitive Research Neuroscience Vrije Universiteit Amsterdam Amsterdam The Netherlands; ^2^ Department of Pathology, Amsterdam Neuroscience Amsterdam UMC Amsterdam The Netherlands; ^3^ Department of Clinical Genetics, Amsterdam Neuroscience Amsterdam UMC Amsterdam The Netherlands

**Keywords:** Aβ, biomarkers, early‐onset Alzheimer's disease, global protein changes, late‐onset Alzheimer's disease, phosphorylated tau, proteomics

## Abstract

**BACKGROUND:**

Alzheimer's disease (AD) occurs primarily as late‑onset (LOAD) and less frequently as early‑onset (EOAD). Its defining pathologies are hyperphosphorylated tau tangles and amyloid beta (Aβ) plaques.

**METHODS:**

We analyzed the proteomes of 115 *post mortem* temporal lobe samples by mass spectrometry and searched with a dedicated AD spectral library including tau post‑translational modifications and Aβ isoforms to examine global protein changes in LOAD and EOAD.

**RESULTS:**

AD tissues showed mitochondrial and synaptic pathway downregulation and immune and small‑molecule metabolic process upregulation, with EOAD exhibiting larger fold changes. AD biomarkers were elevated, and two multi‑phosphorylated tau peptides (p‑tau231/p‐tau235 and p‐tau231/p‐tau235/p‐tau237) were detected predominantly in AD. Aβ was present in 45% of cognitively unimpaired elderly controls, with subtle proteome changes resembling an early stage of neurodegeneration.

**DISCUSSION:**

EOAD appears more aggressive. Tau p‐tau231/p‐tau235 and p‐tau231/p‐tau235/p‐tau237 hold promise as novel AD biomarkers. Aβ’s detection in cognitively unimpaired elderly controls precedes clinical AD symptoms.

## Highlights

- Our proteomics approach to analyzing AD brains confirms the elevated AD biomarkers such as p‐tau181, p‐tau217, p‐tau231, and tau MTBR‐tau243 and the pathophysiological changes in AD brains as implicated by their proteome alterations.
- We report several multi‐phosphorylated tau peptides as potential AD biomarkers.
- We demonstrate shared proteome changes with stronger alterations in EOAD versus LOAD.
- Aβ‐positive cognitively unimpaired individuals exhibit preclinical or resilient neurodegeneration.

## 1 BACKGROUND

Alzheimer's disease (AD) is the most commonly occurring neurodegenerative disease, accounting for 60% to 80% of all dementia cases. Globally, over 55 million people suffer from dementia. The progression of AD might span decades, first without clinical manifestation and later involving memory impairments, cognitive decline, and eventually complete dependence and death.^1^ Currently, there is no cure for AD. Despite the identification of many risk factors, the precise underlying mechanism leading to AD still remains largely unclear.^2^

Neuropathological hallmarks of AD are the accumulation of amyloid beta (Aβ) aggregates in plaques^3^ and the hyperphosphorylated tau presented intra‐neuronally as neurofibrillary tangles (NFTs).^4^ Plaques are protein deposits of Aβ isoforms derived from abnormal cleavages of the amyloid precursor protein (APP). Aβ is prone to aggregate‐forming oligomers and, eventually, high‐density plaques. The oligomers show high cellular toxicity.^5^, ^6^, ^7^ NFTs are intracellular aggregates formed by bundles of extensively post‐translationally modified tau protein.^8^, ^9^ This AD pathology is accompanied by more general features of neurodegeneration including synapse and neuronal loss, neuroinflammation, and reactive astrogliosis,^10^ as well as aberrant proteostasis, cytoskeletal abnormalities, altered energy homeostasis, and DNA and RNA defects.^11^ The presence of Aβ may serve as an initiator of pathological processes that lead to neurodegeneration,^12^ but Aβ plaques are also commonly found in people without AD symptoms,^12^ especially in the aged population. Instead, the spread and intensity of NFTs in the brain correlate better with AD progression and clinical manifestation,^13^, ^14^ which may indicate a central role of NFTs in disease progression.^15^ AD manifests mostly as late‐onset AD (LOAD) sporadic cases in the older population; however, there are also individuals who develop symptoms before the age of 65, often classified as early‐onset AD (EOAD). EOAD is usually caused by familial mutations and follows an atypical and aggressive clinical course.^16^

The examination of global protein changes in the AD brain may yield valuable insights into the disease mechanism. Proteomics has emerged as a leading technique for quantifying changes in tissue proteomes and has been extensively applied in the analysis of AD brain tissues, including early to advanced stages and across patient subtypes.^17^, ^18^, ^19^, ^20^, ^21^, ^22^ Proteomics studies of AD brain tissues of different stages have shown different biological processes to subsequently occur in AD. They identified additional molecular changes relevant to AD pathophysiology that are not reflected at the mRNA level.^22^ Interestingly, recent studies on isolated tau aggregates indicated various specific post‐translational modifications (PTMs) associated with different neurodegenerative diseases.^23^, ^24^, ^25^, ^26^ Although three tau phospho‐peptides, p‐tau181, p‐tau217, and p‐tau231, are widely used as AD biomarkers in plasma,^27^ PTMs are relatively understudied in large‐scale proteomics analysis. In particular, defining age‐specific PTMs could lead to new, more specific biomarkers for AD.

Current bottom‐up proteomics workflows, in particular those using data‐independent acquisition (DIA) mode,^28^, ^29^ routinely quantify several thousand proteins in a single liquid chromatography‐mass spectrometry (LC‐MS) run based on the detection of their tryptic peptides. This approach allows an excellent quantitative analysis between samples for a large range of proteins. However, tryptic digestion of Aβ isoforms generates largely semi‐tryptic peptides, which cannot be matched to a standard FASTA library, as is widely used for peptide identification, and therefore these would not be detected. Similarly, AD‐associated hyperphosphorylated tau peptides are generally not included in such libraries and therefore would not be recognized in a conventional database search, leading to their exclusion in the analyzed data.

In this study, we built a comprehensive spectral library including tau tryptic peptides with PTMs and Aβ isoforms and used it to search the proteomics raw data generated from the DIA run of brain temporal lobe extracts. The proteome changes in AD including the tau phospho‐peptides and Aβ isoforms aligned well with the AD pathology, with EOAD as a more aggressive form. Aβ was detected in about half of the cognitively unimpaired elderly controls with subtle proteome changes that partly resembled the changes of biological processes in AD.

## 2 METHODS

### 2.1 Case selection

Tissues from AD cases and cognitively unimpaired individuals were provided by the Netherlands Brain Bank (NBB, https://www.brainbank.nl). All brain tissues were collected from donors who had provided written informed consent for brain autopsy and the use of brain tissue and clinical information for research purposes. The brain donor program of the NBB was approved by the local medical ethics committee of the Vrije Universiteit Amsterdam (VU) medical center. Forty‐two cognitively unimpaired controls and 73 AD individuals were used in this study. The clinical status of each subject was documented prior to brain donation to ascertain the cognitively unimpaired or AD status. The AD cases were classified in EOAD (age 56 to 70, 25 cases) and LOAD (age 85 to 96, 48 cases), and their age‐matching cognitively unimpaired controls were classified as Early Age (EA, ages 56 to 70, 11 cases) and Late Age (LA, age 85 to 96, 31 cases), respectively. The clinical covariates were not considered for the analysis.

**RESEARCH IN CONTEXT**

1. **Systematic review**: Using PubMed on omics‐ studies of AD we found a number of studies that described the global changes of mRNA/proteins in AD brains. The hallmarks of AD, i.e., post‐translational modification of tau and Aβ isoforms, were not interrogated in ‐omics studies.
2. **Interpretation**: Our findings describe the proteome changes in AD brain, reveal known and novel PTMs of tau and Aβ fragments, and demonstrate quantitative differences between EOAD and LOAD. We found minor proteome changes in Aβ‐positive cognitively unimpaired individuals, the biological process of which is consistent with early pathology of neurodegeneration.
3. **Future directions**: We identified a number of novel potential AD biomarkers; future studies are needed to evaluate their diagnostic and prognostic performances to classify AD patients.

### 2.2 Library generation

Brain tissues were potter extracted in 1% n‐dodecyl‐β‐D‐maltoside (DDM) and centrifuged in Eppendorf tubes for 10 min at 18,000 × g, 4°C. The soluble and insoluble fractions were separately solubilized in Tricine sample buffer (pH = 8.45) and run on a 10% Tricine gel (Novex Tricine Mini Protein Gel from Invitrogen/Thermo Fisher Scientific, USA) that separated proteins down to 2.5 kDa. The gel lane was stained with colloidal Coomassie Blue G‐250, cut into 26 fractions, destained, and then digested by trypsin/Lys‐C (Promega, USA), as described previously.^30^

The peptide solution was transferred to an Evotip and run on a 15 cm × 75 µm, 1.9 µm Performance Column (EV1112, EvoSep) using the Evosep One LC system with the 30‐samples‐per‐day program. Peptides were electro‐sprayed into a timsTOF Pro 2 mass spectrometer (Burker, Germany) and analyzed with Data‐Dependent Acquisition with Parallel Accumulation‐Serial Fragmentation (ddaPASEF). The MS scan was between 100 and 1700 m/z. The Tims settings were 1/Ko from start to end between 0.6 and 1.6 Vs/cm^2^, ramp time 100 ms, accumulation time 100 ms, and ramp rate 9.42 Hz.

The raw data were analyzed by Fragpipe version 22.0^31^ with variable modifications of Ser/Thr/Tyr phosphorylation, Lys ubiquitination, Lys acetylation, Lys methylation/di‐methylation/tri‐methylation, and Met oxidation, using trypsin digestion with two missed cleavages, precursor mass tolerance of 10 parts per million (ppm), and fragment mass tolerance 20 ppm. The FASTA files for database search consisted of UP000005640_9606.fasta from UniProt reference proteomes 2023‐03. We created several artificial precursors that contain known Aβ isoforms and specific peptides for tau isoforms and incorporated these into the FASTA for the database search. The validation tools used in Fragpipe were MSBooster with predict Retention time (RT) and predict spectra for rescoring using deep learning prediction, Peptide‐Spectrum Match (PSM) validation with Percolator, PTM site localization with PTMprophet, and protein inference with proteinprophet. A spectral library was generated with EasyPQP version 0.1.50.

### 2.3 Sample preparation and LC‐MS analysis

Fresh frozen tissue of the middle temporal lobe was cut into 10‐µm‐thick sections using a cryostat and mounted on polyethylene naphthalate‐membrane slides (Leica, Herborn, DE). Sections were fixed in 100% ethanol for 1 min and stained using 1% (w/v) toluidine blue in H_2_O (Fluka Analytical, Buchs, Switzerland) for 1 min. Laser micro‐dissection (LMD) was performed using a Leica AS LMD system (Leica, Germany) to isolate 0.5 mm^3^ of gray matter tissue and collected in 30 µL 3 M‐PER lysis buffer (Thermo Fisher Scientific, USA) in 0.5‐mL Eppendorf PCR tubes and stored at −80°C until further use.

Samples were heated to 95°C for 5 min and loaded on 10% Bis/Tris‐polyacrylamide gels and run into the gel for 15 min at 80 V using 1.5 M Tris/Glycine SDS running buffer at pH 8.3. Gels were fixed overnight and stained with colloidal Coomassie Blue G‐250 before samples were cut out and small gel pieces of about 1 mm^3^ were placed in 96‐well Nunc filter plates (Thermo Fisher Scientific, USA). Destaining, trypsin digestion, and peptide extraction were done as described. Tryptic peptides were analyzed in an Evosep‐timsTOF pro2 setup as described in the library generation section above, with the exception that diaPASEF was used.^32^

### 2.4 Data analysis

The raw data were searched with DIA‐NN 1.9.2,^33^ using the dedicated spectral library as described above. Protein inference was set to Isoform IDs. Match‐between‐runs was activated, with no shared spectra.

Mass Spectrometry Downstream Analysis Pipeline (MS‐DAP) 1.0.5^34^ was used for downstream analysis of the DIA‐NN results and to perform differential expression analysis. In MS‐DAP settings, filter_min_detect was set to 3, filter_fraction_detect was set to 0.5, filter_fraction_quant was set to 0.5, Variance Stabilizing Normalization (VSN) and modebetween_protein was used to normalize the data. MSqRob was used for the differential abundant analysis, with *q* < 0.05 as a filter for the significant alteration of proteins between the two contrasting groups.

The overexpression Gene Ontology (GO) analysis of the regulated proteins was performed in ShinyGO,^35^ with false discovery rate (FDR) cutoff at 0.05 and minimum pathway size of 10; up‐ and downregulated proteins were separately analyzed. A hierarchical clustering tree generated by ShinyGO summarized the correlation among significant pathways. To construct a bubble plot of GO terms for the comparison across the groups, we selected 24 GO terms from branches of the tree as representative examples of the pathways with many shared genes. GO terms with adjusted *p* < 0.05 were included in the GO bubble plot.

Gene set enrichment analysis was performed in GOAT^36^ with the GO database (2025‐1‐1), gene score type “effect size,” gene set size between 10 and 1500, and *p* value adjustment with FDR at a threshold of 0.05. The heatmap was set at 6 cluster count.

The analysis results of box plots for tryptic peptides by t‐test, the receiver operating characteristic curve plot (using the pROC R package), the correlation plot of peptide intensities between tau, and PTMs to Aβ were generated using the CNSknowall platform (https://cnsknowall.com), the cluster volcano plot by OmicStudio.^37^ A schematic diagram of tau with PTMs were drawn in the web server from IBS 2.0.^38^ Data are available via ProteomeXchange with the identifiers PXD066886, PXD067113, PXD067114, and PXD067115.

## 3 RESULTS

**FIGURE 1:**
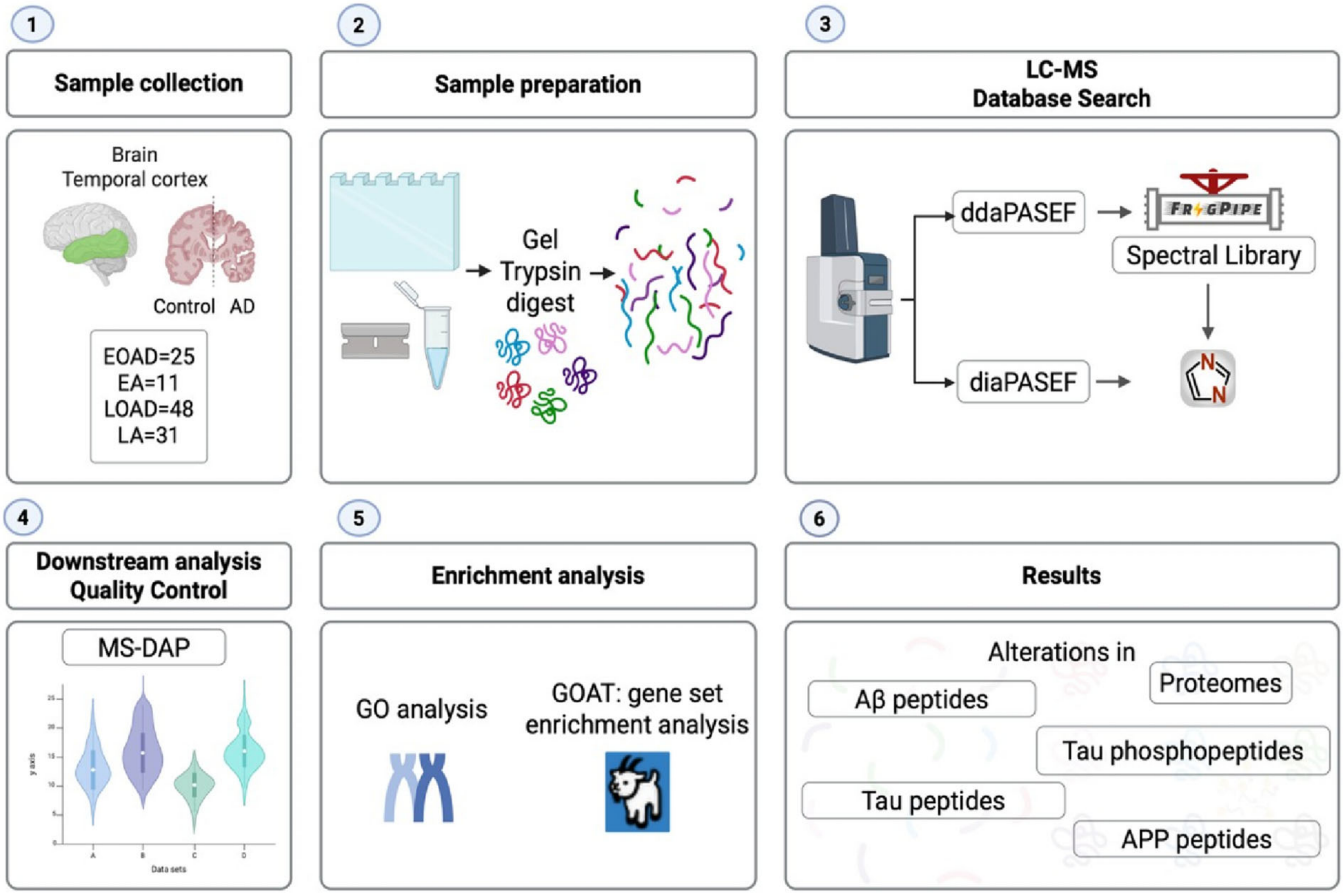
Schematic overview of experimental workflow. (1) Samples were brain slices from *post mortem* brain temporal lobe from EOAD and LOAD patients and their age‐matched cognitively unimpaired EA and LA control groups. (2) Samples were separated on gel and digested by trypsin. (3) The tryptic peptides were analyzed by LC‐MS. In the first instance, a dedicated spectral library was built. Each sample run on gel was cut into 26 fractions and individually digested and subjected to ddaPASEF. All ddaPASEF data were collectively searched with FragPipe, which included sites of phosphorylation and ubiquitination to build a dedicated spectral library. For the analysis of the EOAD, LOAD, and EA and LA groups, a sample was run briefly into the gel and cut as a single slice that contained all the proteins. After digestion, peptides were subjected to diaPASEF analysis. Raw MS data were analyzed with DIA‐NN using the dedicated spectral library. (4) Downstream analysis and quality control was performed with MS‐DAP. (5) For functional characterization of global changes between EOAD and EA, LOAD and LA, EA and LA, and EOAD and LOAD, we performed over‐representation GO analysis with ShinyGO. To reveal the minor changes between EOAD and LOAD, we performed gene set enrichment GO analysis with GOAT. (6) Quantified proteins were used for the analysis of alteration in proteomes between groups. In addition, analyses were carried out at the level of tryptic fragments of Aβ and tau including PTMs, which were largely missed in previous proteomics studies. ddaPASEF, Data‐Dependent Acquisition with Parallel Accumulation‐Serial Fragmentation.

We analyzed the *post mortem* temporal lobe samples from EOAD, EA (early age, age‐matched cognitively unimpaired control group to EOAD), LOAD and LA (late age, age‐matched cognitively unimpaired control group to LOAD) by a dedicated library‐based DIA proteomics analysis. This approach allows the characterization of phosphorylation and ubiquitination sites on the tryptic peptides, and the identification of various Aβ‐truncated forms of which many are semi‐tryptic peptides not detected by regular DIA‐based analysis. A schematic overview of the experimental workflow is shown in Figure 1.

### 3.1 Construction of an AD‐specific peptide spectral library with PTMs

We performed a Data‐Dependent Acquisition (DDA)‐based experiment to build a spectral library from 104 LC‐MS runs. As in DDA mode single precursors for fragmentation and identification are selected, the low complexity of the spectrum allows the identification of peptides with multiple PTMs. Variable modifications for Lys ubiquitination, acetylation, methylation/bi‐methylation/tri‐methylation, and Ser/Thr/Tyr phosphorylation were included. We created several artificial precursors that contain known Aβ isoforms and specific peptides for tau isoforms and incorporated these into the FASTA for the database search in FragPipe (Table S1). Together, a spectral library with 118,402 peptides and 9046 protein groups was created. Data are available via ProteomeXchange with the identifier PXD066868.

**FIGURE 2:**
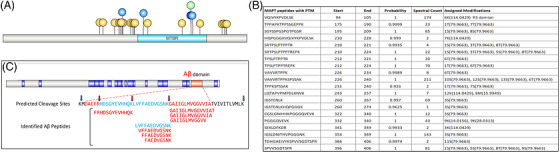
Identification of tau tryptic peptides, including PTMs and Aβ tryptic peptides, by ddaPASEF analysis from cognitively unimpaired controls and AD patients. (A) Tau precursor (2N4R isoform) with the identified post‐translational modifications and the coverage of the precursor sequences. The MTBR domain is highlighted in pale blue. The sites of PTMs shown were phosphorylation (yellow circle), di‐methylation (dark blue circle), methylation (green circle), and ubiquitination (blue circle). (B) Annotation of the tau tryptic peptides with PTMs. These peptides were matched with probability >0.99, with the exception of two peptides that were matched at 0.96 and 0.92 that have been counted one and two times, respectively, implicating their lower abundance. (C) Identified App peptides (labeled in dark blue boxed domains), sequence of Aβ, and identified peptides as generated by trypsin digestion from the amino, central, and carboxyl termini of Aβ. With the exception of LVFFAEDGSK, the identified peptides were the semi‐tryptic digested products. The predicted tryptic cleavage sites within Aβ are indicated by red arrows, the cleavage sites preceding and proceding Aβ by blue arrows. The identifid Aβ‐specific tryptic peptide sequences are lableled in red, the tryptic peptides that are shared between Aβ and App in light blue. ddaPASEF, Data‐Dependent Acquisition with Parallel Accumulation‐Serial Fragmentation.

The coverage of PTMs in tau (represented here by the most commonly cited isoform, 2N4R) is shown (Figure 2A), which includes 19 phosphorylation, three ubiquitination, and single mono‐methylation and di‐methylation sites with high confidence of >0.9 probability.

Regular proteomics data analysis typically searches peptides corresponding to the trypsin cleavage sites with a carboxyl terminal Arg or Lys residue, except for the single peptide from the precursor located at the carboxyl terminal. On the other hand, Aβ isoforms are not full tryptic peptides ending with non‐Arg/Lys carboxyl termini and thus are generally not detected in proteomics studies. To address this, we included a custom set of protein sequences in the database search that covered previously reported Aβ isoforms. More specifically, we created artificial precursors that contain peptide sequences corresponding to the previously reported Aβ isoforms, placed these at the carboxyl termini of the virtual precursors (Table S1), and incorporated these into the UniProt human FASTA file for database search. Using this strategy, we detected peptides that were likely derived from Aβ1‐40 and 1‐42, namely, Aβ29‐40 and 29‐42 (Figure 2B). In addition, we matched a peptide to an extended form of Aβ29‐43. A series of shorter semi‐tryptic peptides corresponding to Aβ18‐28, 19‐28, 20‐28, and 4‐16 were also detected.

### 3.2 Proteomics analysis of EOAD and LOAD identifies EOAD as the more aggressive form of AD

**FIGURE 3:**
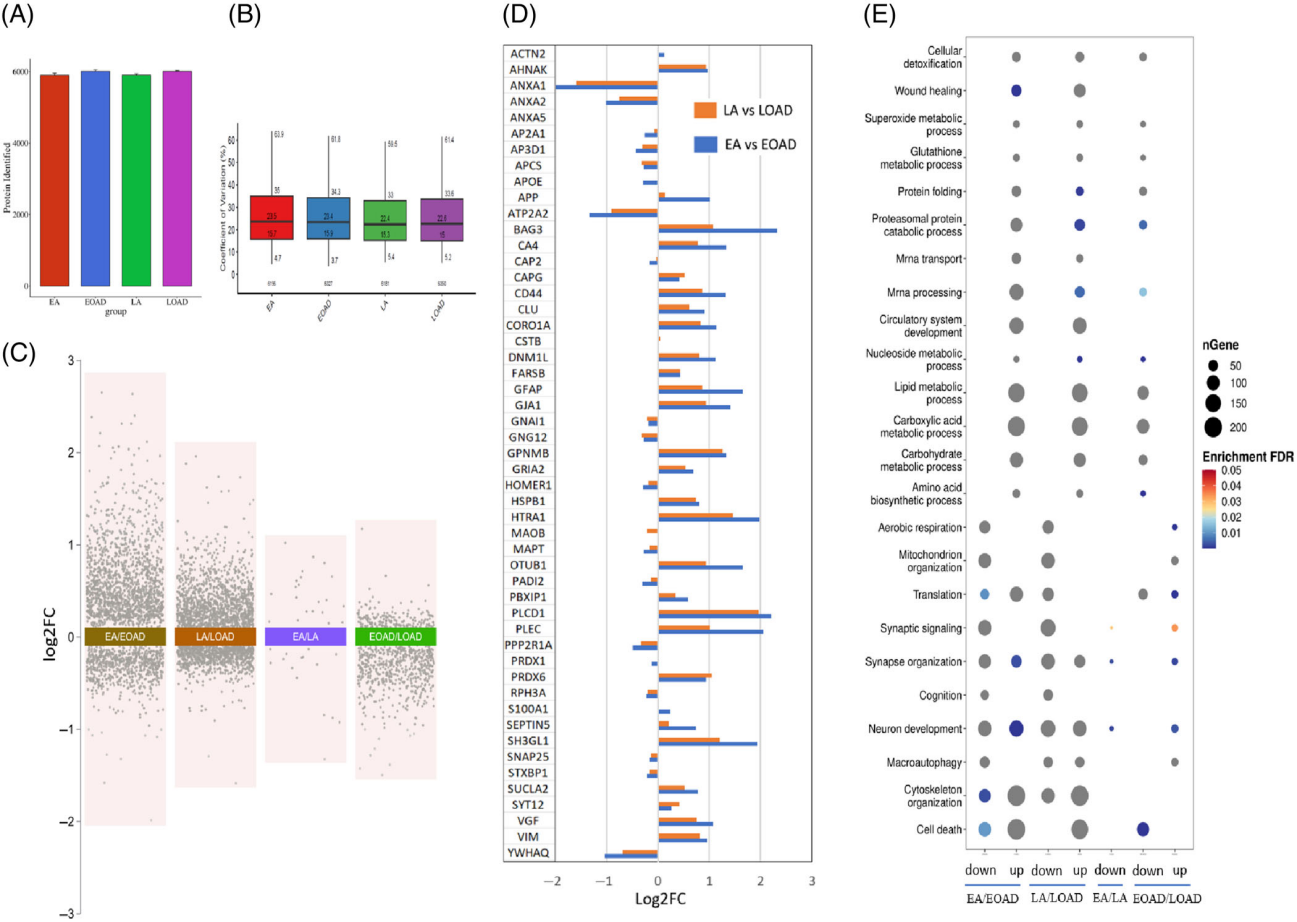
Proteomics analysis of temporal lobe extracts from EOAD and LOAD and their age‐matched cognitively unimpaired controls EA and LA. (A) Approximately 6000 protein groups were identified for each of the four groups. (B) The coefficient of variation (CoV) of the proteomics measurements for the four groups centers around 20%. (C) Clustered volcano plot showing fold changes for each significant regulated protein from the comparison of EOAD‐EA, LOAD‐LA, EA‐LA, and EOAD‐LOAD. (D) The fold changes of selected proteins. These proteins were compiled as the most common protein changes in AD reported in multple proteomics studies. (E) GO enrichment analysis for the comparisons EOAD‐EA, LOAD‐LA, EA‐LA, and EOAD‐LOAD. Shown are the representative GO terms for specific groups of biological processes. The up‐ and downregulation were separately analyzed. In the EA‐LA comparison, GO analysis of upregulated proteins yielded no GO term; downregulation revealed minor changes in synapse organization and neuron development.

We analyzed the *post mortem* human temporal lobe of 42 subjects without AD pathology versus 73 with pathology, each spanning between two age groups of 56 to 70 and 85 to 96 and with or without AD pathology. Samples were measured by diaPASEF, searched with DIA‐NN using the AD‐dedicated spectral library, and analyzed by MS‐DAP for quality control and downstream data analysis. On average, ∼6000 protein groups were identified per case for EOAD and LOAD and the age‐matched EA and LA (Figure 3A), with an overall coefficient of variation around 20% for all four groups (Figure 3B). The 85 to 96 age group had a larger sample size due to the larger number of cases available in the brainbank.

We contrasted the experimental groups in pairs to reveal the proteome differences due to (1) the AD pathology versus cognitively unimpaired control of the same age, (2) the effect of aging itself, and (3) age‐dependent changes induced by AD that may differentiate the pathological effects between EOAD and LOAD. The differential abundance analysis of the pairwise comparisons of the groups generated by MS‐DAP are shown in Table S2, the fold changes of selected proteins in Table S3, and the GO terms generated by ShinyGO in Table S4.

The cluster volcano plot in Figure 3C shows proteins with significant fold changes at FDR < 0.05. EOAD‐EA comparison yielded significant regulated proteins (2290 proteins), LA‐LOAD comparison yielded 2304 proteins, and EOAD‐LOAD comparison yielded 767 proteins. The differences between EA and LA were minor (36 proteins).

A meta study^21^ examining previous proteomics studies identified the top 54 proteins that are consistently altered in AD. The fold changes of these proteins that were also identified in the present study are shown in Figure 3D, which demonstrates the consistency of the direction of changes as reported in previous studies. Interestingly, the levels of protein fold changes in EOAD (EA/EOAD) were generally higher than those observed in LOAD (LA/LOAD).

To reveal the biological processes that were altered in the AD groups, we performed GO overexpression analysis with ShinyGO. The significantly enriched GO terms for LA‐LOAD (Figure 3E) are in good agreement with previous findings and showed in LOAD the downregulation of mitochondrial processes (mitochondrion organization, aerobic respiration) and chemical synaptic transmission (synaptic signaling, synapse organization, neuron development, cognition), the upregulation of processes involved in mitigating oxidative stress and maintaining cellular homeostasis (cellular detoxification, superoxide metabolic process, glutathione metabolic process, wounding healing, proteasomal process catabolic process, protein folding), small molecule metabolism (lipid metabolic process, carboxylic acid metabolic process, amino acid biosynthetic process, carbohydrate metabolic process), and cell death. Similar findings were obtained in the comparison of EA‐EOAD. We then compared EOAD‐LOAD to reveal their quantitative differences. Compared to EOAD, LOAD showed the upregulation of mitochondrial process and synaptic neurotransmission and downregulation of small molecule metabolism and processes involved in mitigating oxidative stress and maintaining cellular homeostasis. These imply that LOAD was less severely affected than EOAD. The differences for EA‐LA were subtle, with the synaptic neurotransmission process slightly downregulated in LA. This indicates that aging in health controls has a negative impact on nerve cells, but the overall effect is mild compared to AD pathologies.

### 3.3 Quantitative analysis of tau at peptide level reveals AD‐specific modifications and differential peptide abundance

Our spectral library contains many tryptic tau peptides, including those with PTMs characterized at specific sites with >90% confidence (Figure 2A,B). This makes it possible to contrast the four groups at the single peptide level with high confidence and correlating their differential abundance and AD clinical manifestation.

**FIGURE 4:**
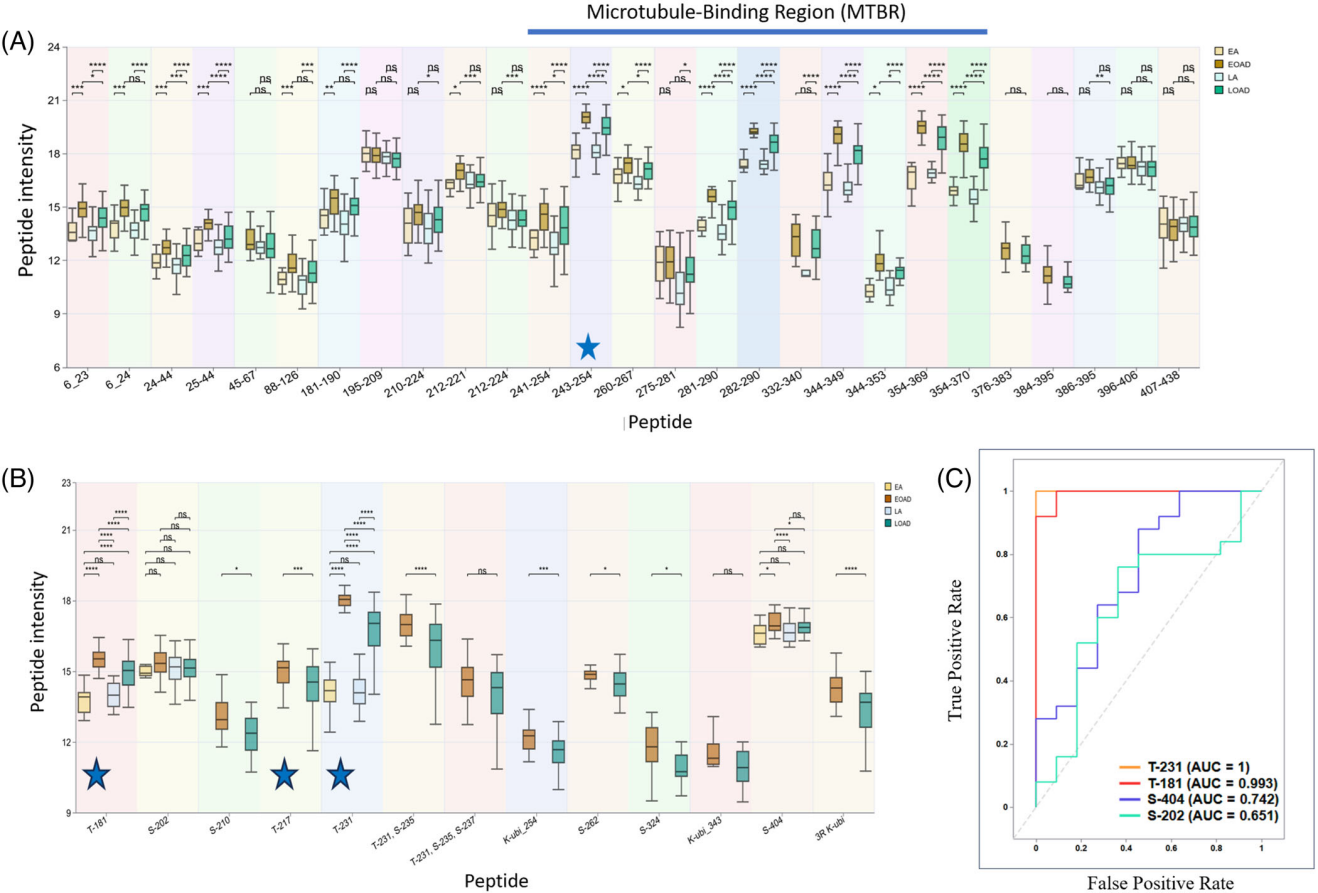
Quantitative analysis of tau tryptic peptides and their post‐translational modifications. (A) Intensity of individual tau tryptic peptides. The AD biomarker MTBR243 is indicated by a star. The MTBR region is underlined by a blue line. (B) Intensities of tau peptides with PTMs. Stars point to peptides corresponding to biomarkers p‐tau181, p‐tau217, and p‐tau231. Peptides that were detected in less than 20% in a group were not included in the statistical analysis. (C) Receiver‐operating characteristic curves for comparison of EOAD and EA showing specificity and sensitivity of peptides that were present in all four groups as shown in (B). Peptides corresponding to biomarker p‐tau181 and p‐tau231 yielded perfect classification of AD from cognitively unimpaired controls. *p < 0.05; **p < 0.01; ***p < 0.005; ****p < 0.001; ns, no significance.

We first analyzed the changes in abundance of tau tryptic peptides in different groups (Figure 4A). The revised criteria for diagnosis and staging of AD^27^ focus on MTBR‐tau243. Here, the MTBR‐tau243 is represented by two peptides, 241‐254 and 243‐254 (Figure 4A), with both showing significant upregulation between AD and cognitively unimpaired controls. The peptides located at the amino terminal, albeit at lower levels than the peptides within the MTBR region, except that MTBR‐tau275‐281 did not change. The peptides 380‐395, 396‐406, and 407‐438 located at the carboxyl terminal were not regulated. Together, this implies that (1) the regulation of the total tau as reported by previous proteomics studies was an average of identified tau tryptic peptides, each of which was differentially regulated, (2) additional potential AD biomarkers may be developed between tau 281 and 395 that have higher fold differences between AD and cognitively unimpaired controls, and (3) the tau peptide fold changes in EOAD are generally higher than those in LOAD.

The comparative analysis of tau peptides with PTMs is shown in Figure 4B. P‐tau181, p‐tau217, and p‐tau231 are the proposed core 1 categories in AD.^27^ As expected, peptides containing p‐tau181 and p‐tau231 (Figure 4B star) show significant upregulation in AD cases, and p‐tau217 was specifically detected in AD (Figure 4B star). Four phospho‐peptides were detected in all four groups (Figure 4B); their receiver‐operating characteristic curves for EA compared to EOAD are shown in Figure 4C. Indeed, p‐tau218 and p‐tau231 gave perfect classification of AD, which is consistent with their status as AD biomarkers. In contrast, the other two phospho‐tau peptides p‐tau202 and p‐tau404 yielded poorer curves, implying that they are basal tau phosphorylation sites regardless of the AD condition. Additional tau peptides with PTMs that deserve consideration are, for example, the double and triple phosphorylated peptides within the tau p‐tau231 region, namely, p‐tau231/p‐tau235 and p‐tau231/p‐tau235/p‐tau237. These peptides were present at relatively high abundance comparable to other modified tau peptides but were below the detection level or present in less than 20% of the cases in the cognitively unimpaired controls. This suggests that they may be candidates for AD biomarkers, on top of the widely used p‐tau231. Finally, the fold changes of p‐tau181, p‐tau217, and p‐tau231 were more pronounced in EOAD than LOAD.

We also detected ubiquitination sites for AD (Figure 4B) at p‐tau254 and p‐tau343 and a peptide at the 3R region (not shown in Figure 1). These peptides were not detected in the cognitively unimpaired controls and, therefore, hold promise as additional AD biomarkers. The raw data from tau tryptic peptides including PTMs are shown in Table S5.

### 3.4 Low correlation of intensities between modified peptides of tau and Aβ at late‐phase dementia

**FIGURE 5:**
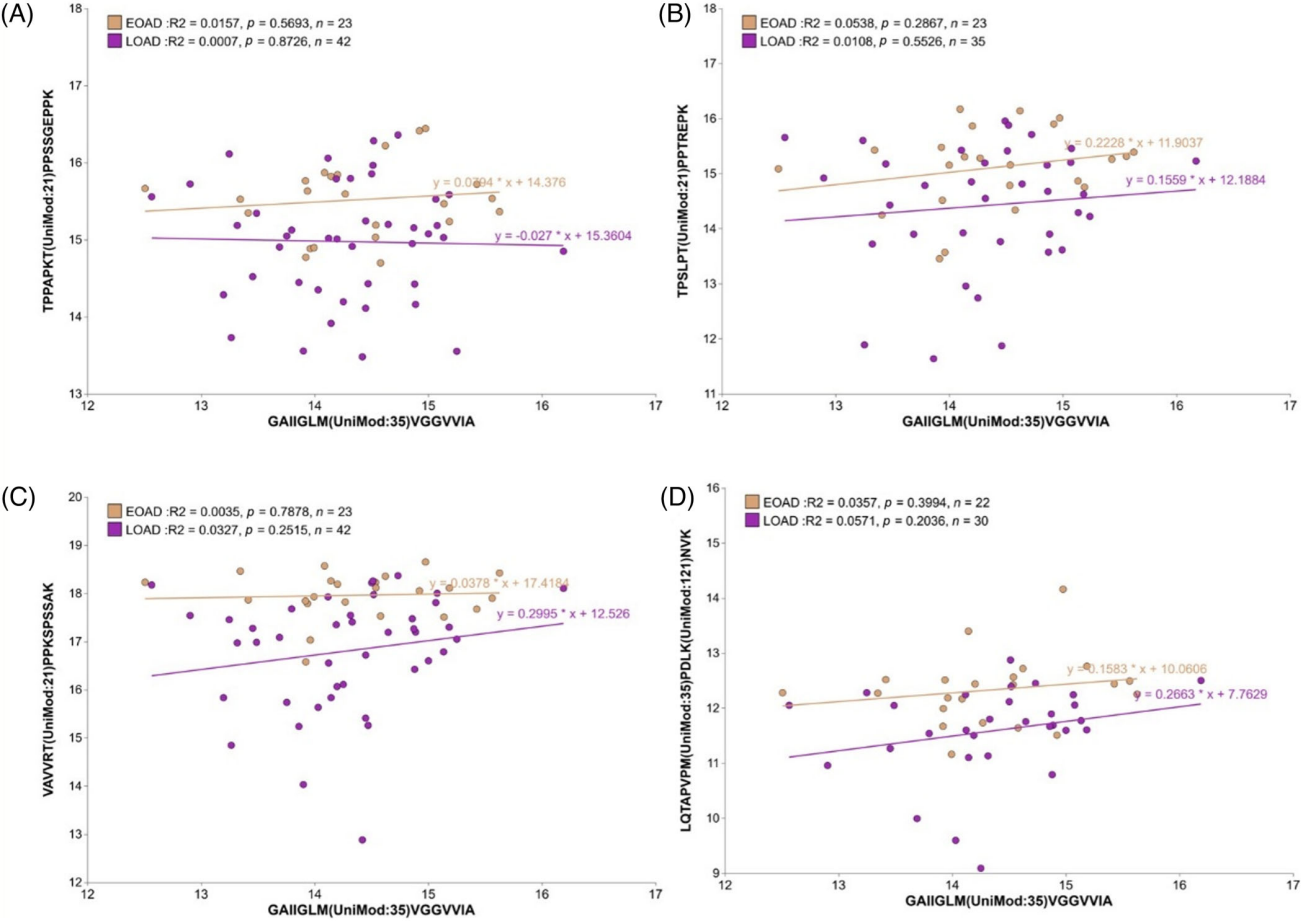
Correlation of peptide intensities between tau (*y*‐axis) and PTMs to Aβ (*x*‐axis). The modified tau peptides shown are (A) TPPAPKTPPSSGEPPK (p‐tau181), (B) TPSLPTPPTREPK (p‐tau217), (C) VAVVRTPPKSPSSAK (p‐tau231), and (D) LQTAPVPMPDLKNVK (ubiquitination‐254). *X*‐axis, Aβ1‐42 as represented by its tryptic peptide Aβ29‐42 GAIIGLMVGGVVIA. *N*, number of cases used for regression lines. uniMod:35 represents M‐oxidation, uniMod:21 represents T(S/Y)‐phosphorylation, and uniMod:121 represents K‐ubiquitination.

Current plasma AD biomarker assays focus mainly on tau phosphorylation at tau residues p‐tau181, p‐tau217, and p‐tau231 and the ratio of Aβ40/42. It has been suggested that different phosphorylation sites on the tau protein are affected at varying times^39^ during the development of AD and subsequent to amyloidosis. To reveal whether there was a link between the quantities of tau modifications and Aβ, we plotted the normalized peptide intentisities of tau with PTMs versus Aβ1‐42 as represented by its tryptic peptide Aβ29‐42. Figure 5 shows the correlation of Aβ and AD upregulated tau peptides LQTAPVPMPDLKNVK (ubiquitination‐254), p‐tau181, p‐tau217, and p‐tau231. The low *R* ^2^ and high *p* value indicate that the intensities of tau peptides were randomly distributed regardless of the Aβ load. In late AD, the peptides had probably already plateaued presumably due to the extensive degeneration resulting in the loss of their association. Tau levels stop increasing in late AD because severe neuronal loss depletes the cells that produce tau, causing their levels to stabilize despite the ongoing disease progression. The raw data for Aβ and tau peptides are shown in Table S6.

### 3.5 Presence of Aβ in LA correlates with minor AD‐type proteome change

**FIGURE 6:**
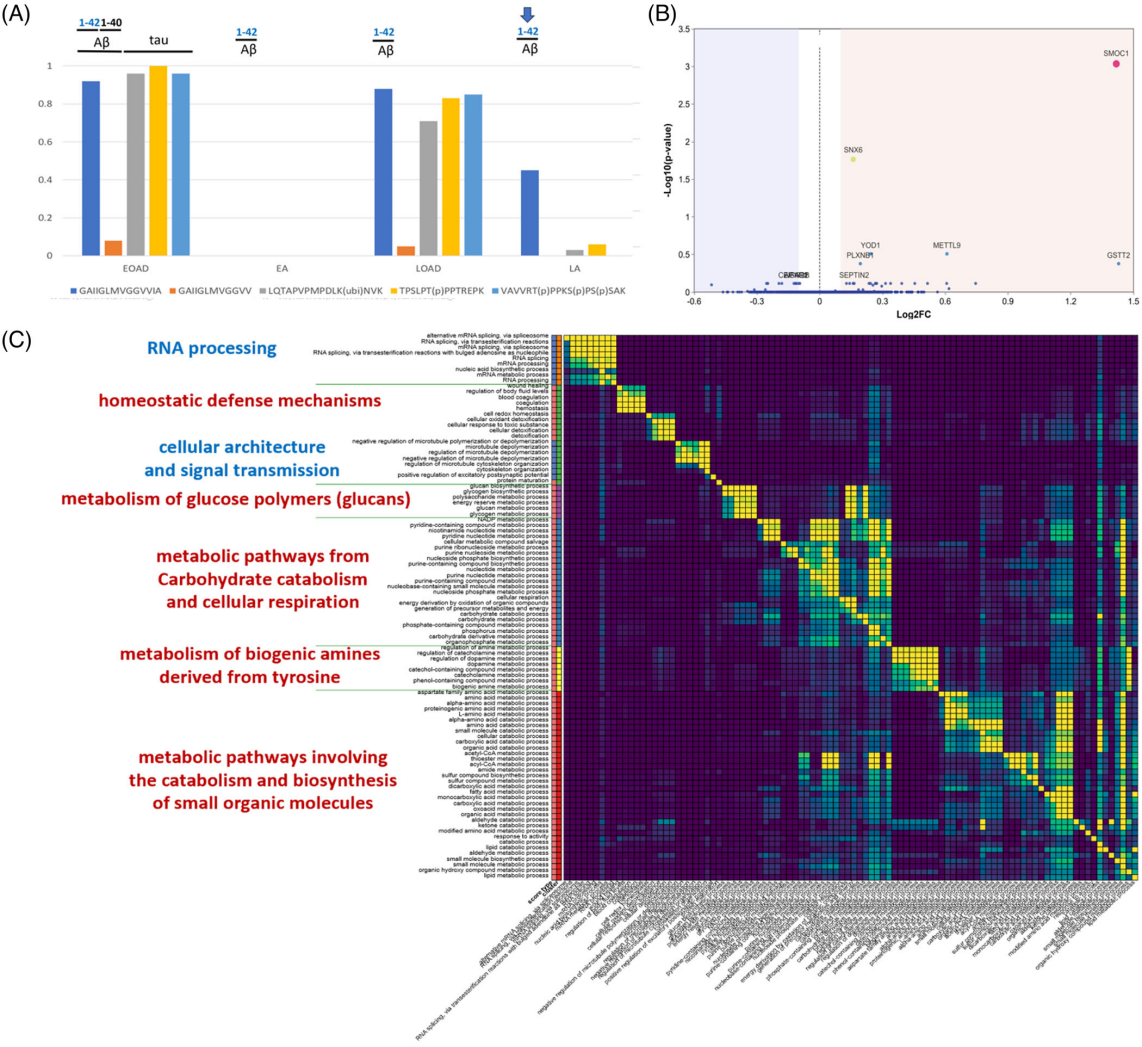
Aβ occurrence in the four AD and control groups and its specific effects on the proteome of LA. (A) Frequency of occurrence of Aβ and tau tryptic peptides in EOAD, EA, LOAD, and LA; Aβ29‐40 (representing Aβ1‐40), Aβ29‐42 (representing Aβ1‐42), and three tau peptides with post‐translational modification of phosphorylation (*p*) or ubiquitination (ubi) on preceding amino acid S/T and K, respectively. Aβ was found in 45% of the LA cases (highlighted with a blue arrow) and none in EA cases. The sequences of the peptides are shown in the lower panel. *Y*‐axis, frequency of occurrence. (B) Volcano plot for comparison of LA with and without Aβ. The differences were subtle with two proteins significantly upregulated in LA with Aβ. (C) Heatmap of biological processes generated by gene set GO enrichment analysis (GOAT) for comparison of LA with or without Aβ. The upregulated clustered biological terms in the Aβ‐containing group are labeled red, the downregulated ones in blue.

The aggregation of Aβ in the brain is one of the hallmarks of AD.^5^, ^15^ Notably, Aβ is also found in cognitively unimpaired individuals, in particular in the aged population. However, the presence of Aβ alone is not sufficient for neurodegeneration. Figure 6A shows the ratios of cases with tryptic peptide Aβ29‐42 and 29‐40 (corresonding to the carboxyl terminal of Aβ1‐42 and Aβ1‐40) and tau tryptic peptides with ubiquitinaton or phosphorylation. Aβ29‐42 was present abundantly in the AD groups, but not in EA. Aβ29‐40 was present in a few AD cases. Interestingly, 45% of the aged cognitively unimpaired controls in LA contained Aβ29‐42, but the majority of them were not associated with the presence of ubiquitinated or phosphorylated tau peptides, which is in contrast to AD, which in most cases contains both Aβ29‐42 and ubiquitinated and phosphorylated tau peptides.

Comparison of cognitively unimpaired control cases with and without Aβ revealed only two significant regulated proteins (Figure 6B, Table S7), SMOC1 and SNX6, where SMOC1 was highly enriched in Aβ‐containing cases. This low number of regulated proteins implies that the presence of Aβ alone might not have a negative effect on the brain, which is in agreement with their cognitive unimpairment. However, a pitfall of the over‐representation GO enrichmnt analysis is that it is highly stringent (in our study, FDR < 0.05). It identifies the strong‐effect genes driving the main pathways but that may not reveal the probable subtle differences between LA with and without Aβ.

Gene set enrichment GO analysis is considered to be the method of choice for detecting subtle differences. Here, we used a gene set enrichment GO analysis platform, GOAT.^36^ GOAT is a parameter‐free algorithm for gene set enrichment analysis of pre‐ranked gene lists. It takes into account the fold changes of all proteins and matches them to the gene sets from each biological process annotated in the GO. The significantly regulated GO terms analyzed with FDR were clustered into a heatmap format (Figure 6C). The complete output from GOAT analysis including biological processes, cellular compartments, and molecular functions are presented in Table S8.

For LA with Aβ we find the upregulation of homeostatic defense mechanisms and the small molecule metabolism and downregualtion of cellular architecture and signal transmission, as well as RNA processes (Figure 6B). These GO terms shared similarities with those of AD patients (Figure 3E), which suggests that the presence of Aβ alone may be responsible for initiating the early phase of AD processes.

## 4 DISCUSSION

Proteomics has been extensively applied in the analysis of AD brain tissues, including early to advanced stages and across patient subtypes.^17^, ^18^, ^19^, ^20^, ^21^, ^22^ However, the tryptic peptides of Aβ isoforms and the post‐translationally modified tau are largely not identified because they are not included in the FASTA file used for database search. To circumvent this problem, previous studies used specific tryptic surrogate peptides (LVFFAEDVGSNK, Aβ17‐28) derived from Aβ^40^ or parallel reaction monitoring of synthetic peptides^41^, ^42^ for identification and quantitation. To analyze phospho‐proteomes in AD, classic phospho‐peptide enrichment strategies were employed prior to LC‐MS analysis.^43^, ^44^ Here, we built a spectral library that included Aβ isoforms and tau peptides with PTMs. We revealed that p‐tau181, p‐tau217, and p‐tau231 showed significant upregulation in AD. This aligns well with typical AD pathology and indirectly validates the accurate assignment of peptide identities. Our approach using the dedicated library for quantitative proteomics is instrumental for the simultaneous quantification of the two AD hallmarks, tau peptides, including those with PTMs, and Aβ isoforms, which were not included in previous AD proteomics studies.^21^, ^45^, ^46^, ^47^, ^48^

AD starts with an asymptomatic (preclinical) stage and progresses through mild cognitive impairment and, ultimately, to dementia.^1^, ^27^ By comparing the proteomes of AD patients with those of age‐matched cognitively unimpaired controls, we revealed massive proteome changes (2290 proteins for EA‐EOAD, 2304 proteins for LA‐LOAD) showing significant differences at 5% FDR. GO analysis showed the changes of GO terms typically associated with AD pathology, as reported previously^11^, ^19^, ^21^, ^22^ and in accordance with the integrative multi‐modal analyses that converge on four major axes of AD pathology – mitochondrial/metabolic dysfunction, synaptic dysfunction, neuroinflammatory and immune‐lipid activation, and proteostasis impairments.^49^ The proteome changes between EOAD and LOAD brains were also extensive, with 767 proteins showing significant differences. Regression analysis of the significantly changed proteins revealed similarities between EOAD and LOAD. This is in line with a previous study that showed a similar pathophysiology of autosomal‐dominant AD and LOAD.^50^ Nevertheless, protein fold changes in EOAD, as well as the currently widely used AD biomarkers p‐tau181, p‐tau217, p‐tau231, and MTBR‐tau243,^27^ were generally higher than those found for LOAD. Furthermore, the more pronounced changes of biological processes in EOAD include decreases in mitochondrial processes and synaptic neurotransmission, increases in detoxification, carboxylic acid metabolic processes, and activation of the immune system that together point to EOAD as a more aggressive form of AD.^50^ This is in line with the clinical manifestation of EOAD in which cognitive and clinical functions tend to decline more rapidly in these younger patients.^16^, ^51^ A recent meta‐analysis revealed that EOAD differed from LOAD in baseline cognition, cognitive decline, and survival time but otherwise had clinical characteristics similar to those of LOAD.^52^ There are also differences between EOAD and LOAD; in particular, EOAD has a lower abundance in (macro)autophagy. It is suggested that the pathophysiology of AD may involve impaired autophagy that is manifested as a reduced ability to clear abnormal protein aggregates.^53^, ^54^, ^55^ This may underlie the lower clearance ability in EOAD, leading to a higher load of Aβ1‐42 and p‐tau181, p‐tau217, and p‐tau231, as well as an increase in neuronal death.^55^

We revealed that Aβ 29‐42 was identified in both EOAD and LOAD, as well as 45% of the aged cognitively unimpaired controls. This agrees with previous findings showing that Aβ1‐42 (represented by the tryptic peptide Aβ29‐42) is the prevalent form in AD^7^ and the ratio of Aβ 42/40 is an established AD biomarker.^27^ It is also in line with the observation that many of the pathologies typically associated with neurodegenerative diseases such as AD are more prevalent with increasing age in cognitively unimpaired individuals.^56^ However, it remains unclear whether the proteoforms associated with these pathologies are similar to those found in AD cases or whether the composition of these proteoforms differs between individuals with AD and those who are cognitively unimpaired.

We quantified many tau tryptic peptides. While all the tryptic peptides are derived from tau, several tau tryptic peptides showed differential abundance in AD cases, especially upregulation within two regions of 241‐267 and 261‐370 in MTBR.^25^ Indeed, MTBR‐tau243 is known as a valid biomarker of AD.^27^ On the other hand, peptides at the carboxyl terminal of 386‐407 did not show significant difference. In accordance with the more aggressive nature of EOAD, the upregulated tau tryptic peptides in EOAD also exhibited generally higher fold changes than those of LOAD. In addition to peptides within MTBR, the tau tryptic peptides located at the amino terminal have been found with predictive value as prospective plasma AD biomarkers of cognitive decline and neurodegeneration.^57^, ^58^ In our study, the peptides located at the amino terminal were indeed upregulated in AD cases, but their fold changes were lower than those located within the MTBR. What causes the differences in abundance of the tau tryptic peptides is not clear. It may be contributed by the differences in the structure of the tau aggregates in AD with a subsegment of the MTBR domain, as demonstrated by cryogenic electron microscopy studies.^23^

Hyperphosphorylated tau is a hallmark of AD pathology.^1^, ^23^, ^27^ P‐tau181, p‐tau217, and p‐tau231 were proposed as core biomarkers of AD.^27^ In AD plasma, p‐tau181 and p‐tau217 were found to be independently associated with both plaques and tangles, whereas p‐tau231 selectively associated with plaques.^59^ P‐tau181 and p‐tau217 were found to become abnormal in the presence of Aβ accumulation, as indicated by Aβ positron emission tomography (PET), before the detection of tau pathology using tau PET^60^; in particular, plasma p‐tau217 has been shown to have predictive value for cognitive decline in patients with preclinical AD.^61^ Furthermore, higher levels of p‐tau181 and glial fibrillary acidic protein were linked to faster cognitive decline.^62^ In our study, we confirmed the over‐representation of p‐tau181, p‐tau217, and p‐tau231 in AD cases. This validated the tissue proteomics of *post mortem* brains as a useful source for biomarker discovery. The double and triple phosphorylated peptides p‐tauT231/S235 and p‐tauT231/S235/S237 were found mainly in AD cases with relatively high intensities. These peptides may serve as novel biomarkers of AD biofluid diagnosis. In addition, we detected ubiquitinated tau tryptic peptides that were mainly present in AD cases. However, we did not observe a correlation between p‐tau and Aβ in AD cases, which might have been caused by their peaking at the late phase of dementia in this study, which could explain the lack of correlation.^63^, ^64^ Our tissue proteomics data may be considered an initial screening for AD biomarkers. Such discovery should be confirmed across large diverse human cohorts, including other tauopathies, to validate specificity. This may form the basis of the development of an ultra‐high‐sensitive assay for the quantitation of low‐abundance p‐tau species in plasma and, eventually, for addressing inter‐lab and inter‐platform variability.^65^

The presence of amyloid plaques starts years before the onset of clinical dementia and has been conceptualized as an asymptomatic preclinical stage of AD.^6^ This is in line with biomarker studies supporting the existence of a significant proportion of older adults who are cognitively unimpaired with Aβ deposition but do show only limited tau pathology in the middle temporal lobe. Moreover, their rates of clinical progression over 5 years are more akin to those without Aβ deposition/tau pathology than with both Aβ deposition and tau pathology.^66^ We first examined the molecular differences in the aged cognitively unimpaired controls with or without Aβ deposition by differential expression analysis and found only two significantly changed proteins, SMOC1 and SNX6. A previous study showed that SMOC1 was found to co‐localize with a subpopulation of amyloid plaques in AD, correlated with plaque load regardless of disease stage, interacted with Aβ, and significantly delayed Aβ aggregation.^67^ We then applied GOAT analysis to evaluate all quantified proteins and their fold changes to determine whether a predefined set of genes showed statistically significant alterations, and we revealed changes that mimicked aspects of AD pathophysiology, including the activation of the immune system, small molecule metabolism, and decreased synaptic neurotransmission. In this respect, Aβ is known to affect synaptic plasticity underlying a decreased dendritic spine, and its interaction with various receptors of microglial cells causing increases in the levels of inflammatory cytokines.^68^ Interestingly, mRNA metabolic processes, rRNA metabolic processes, ribosome biosynthesis, and chromatin organization are downregulated, in contrast to LOAD.

To better understand AD pathology as a disease spectrum, future studies will need to examine cases with heterogeneous presentation and penetrance of clinical symptoms, mixed pathologies, potential disease subtypes, and other associated endophenotypes in different brain regions. Bulk tissue analysis remains invaluable for large‐scale clinical correlation and for hypotheses where the average tissue state is the relevant biological variable, while spatially resolved single‐cell analysis would be indispensable for the unbiased discovery of cell types and states that could reveal the novel microglia, astrocyte, and vascular cell states critical for the initiation and progression of AD.^69^, ^70^, ^71^

## CONFLICT OF INTEREST STATEMENT

The authors declare no conflicts of interest. Author disclosures are available in the Supporting Information.

## CONSENT STATEMENT

All brain tissues were collected from donors with written informed consent for brain autopsy and the use of brain tissue and clinical information for research purposes. The brain donor program of the NBB was approved by the local medical ethics committee of the VU medical center.

## Supporting information



Supporting Information

Supporting Information

Supporting Information

Supporting Information

Supporting Information

Supporting Information

Supporting Information

Supporting Information

Supporting Information
