# Supplementary material for: Proteomics of post mortem brains in early‐ and late‐onset Alzheimer's disease: Unraveling differential Aβ effects and potential AD biomarkers
Source: Alzheimers Dement. 2026 Jul 12;22(7):e71662. doi: 10.1002/alz.71662 (PMC13357694; doi:10.1002/alz.71662)
Supplement: Supplementary file 1 — Supporting Information [file ALZ-22-e71662-s008.docx]

**sTable1. The artificial precursors containing known Aβ isoforms and specific peptides for tau isoforms**

>Abeta_truncation_GVV

DAEFRHDSGYEVHHQKAEFRHDSGYEVHHQKEFRHDSGYEVHHQKFRHDSGYEVHHQKLVFFAEDVGSNKVFFAEDVGSNKFFAEDVGSNKFAEDVGSNKAEDVGSNKEDVGSNKGAIIGLMVGGVV

>Abeta_Trunction_GVVIA

QYTSIHHGVVEVDAAVTPEERGAIIGLMVGGVVIA

>Abeta_GVIAT_MAPTisofoms

STPTAEDVTAPLVDEGAPGKSTPTAEAEEAGIGDTPSLEDEAAGHVTQARAEEAGIGDTPSLEDEAAGHVTQARHVPGGGSVQIVYKPVDLSKVQIVYKPVDLSKGAIIGLMVGGVVIAT

>Abeta_GV_AbetaN3pE

EFRHDSGYEVHHQKGAIIGLMVGGV
